# CircRNAs and Erectile Dysfunction: A Hybrid Review of Possible Interactions

**DOI:** 10.3390/cimb48100994

**Published:** 2026-09-26

**Authors:** Dimitrios Triantis, Christina Dastamani, Paschalis Barboutis, Theodoros Petranis, Christos Michalakis, Dimitrios Mavrakis

**Affiliations:** 1 School of Medicine, Aristotle University of Thessaloniki, 54124 Thessaloniki, Greece; 2 Pathology Laboratory, General Hospital of Karditsa, 43100 Karditsa, Greece; 3 Department of Urology, General Hospital of Karditsa, 43100 Karditsa, Greece; 4 Department of Internal Medicine, General Hospital of Karditsa, 43100 Karditsa, Greece

**Keywords:** circular RNA, circRNA, erectile dysfunction, non-coding RNA, ceRNA, endothelial dysfunction, NO/cGMP/PKG pathway, RhoA/ROCK, NF-κB, miRNA sponge

## Abstract

Erectile dysfunction (ED) is a prevalent condition involving multiple molecular mechanisms. Although non-coding RNAs have emerged as important regulators of gene expression, the role of circular RNAs (circRNAs) in ED remains poorly defined. CircRNAs are characterised by a covalently closed loop structure that confers enhanced stability and enables diverse regulatory functions. This review aims to investigate the potential interactions between circRNAs and the signalling pathways linked to ED. Only two primary studies directly investigating circRNA dysregulation in ED were identified, highlighting the early stage of this field. This hybrid systematic narrative review combines a PRISMA-guided systematic search with a pre-specified, hypothesis-generating narrative synthesis of the broader circRNA literature across key signalling pathways implicated in ED, including the NO/cGMP/PKG, RhoA/ROCK, JNK/c-Jun/Bak, and TNF-α/NF-κB axes. We identify several circRNAs as potential regulators of endothelial dysfunction, inflammation, and smooth muscle contractility. CircRNAs therefore emerge as candidate biomarkers and potential therapeutic targets. Given that direct evidence is currently limited to two animal studies, with no circRNA validated in human tissue or as a biomarker, these molecules should be regarded only as candidates for future biomarker and therapeutic target validation.

## 1. Introduction

Erectile dysfunction (ED) is among the most common male sexual disorders globally; prevalence estimates vary widely depending on the assessment method, age group, and geographic region studied. Studies estimate worldwide ED prevalence to be 13.1–71.2% [1]. Projections made in 1995, for example, estimated that 322 million men worldwide would be living with ED by 2025, which would be more than twice the number of men affected at the time [2]. Apart from the direct sexual effects, ED also has a wide-ranging impact on the mental health of the affected person, partner satisfaction and work productivity [3]. Importantly, it is also established as an independent cardiovascular risk factor, indicating its systemic vascular origin [4].

The causes of ED are multifactorial and historically have been divided into organic, psychogenic and mixed [5]. Organic causes encompass hypogonadism, hypothyroidism, diabetes mellitus, coronary artery disease, Parkinson’s disease, and spinal cord injury among others. Substance abuse and the iatrogenic adverse effects of particular medications are also considered. Psychogenic causes such as depression and various other psychiatric conditions are also considered [5,6]. On a cellular scale, the process of erection and flaccidity is the result of the mutually opposing contractile states of the smooth muscle cells of the corpora cavernosa. The erection process is primarily regulated by the NO/cGMP/PKG pathway, with the absence of erection (flaccidity) sustained through the calcium–calmodulin-dependent stimulation of myosin light chain kinase and the RhoA/ROCK (Rho-associated coiled-coil-containing protein kinase) pathway [7]. Despite the fact that ED can be the consequence of various pathogenic processes [8], the two main vascular processes are a decrease in the endothelial NO bioavailability and the subsequent disturbance of the NO/cGMP/PKG pathway, and a pathological overexpression of the RhoA/ROCK pathway [9].

Historically RNA was considered a simple intermediate in the flow of genetic information. This view has been substantially revised by evidence from multiple non-coding RNA (ncRNA) classes. Non-coding RNAs (ncRNAs), characterised by their inability to encode proteins, are established as key modulators of gene expression occurring at the transcriptional, post-transcriptional, and epigenetic stages [10]. Categorisation is generally determined by the transcript size into small ncRNAs (sncRNAs, <200 nucleotides) and long ncRNAs (lncRNAs, ≥200 nucleotides). In the class of sncRNAs, microRNAs (miRNAs) are a group of short non-coding RNAs that are post-transcriptional regulators of gene expression. lncRNAs are functionally diverse, acting as molecular scaffolds, transcriptional decoys and guides to chromatin remodelling, among other roles [11,12].

Circular RNAs (circRNAs) are a specific category of non-coding RNAs (ncRNAs) with a covalently closed continuous loop configuration formed by back-splicing mechanisms. This covalently closed structure confers marked resistance to exonucleolytic digestion, resulting in increased molecular stability [13]. Furthermore, circRNAs display marked tissue-specific and developmental stage-specific expression profiles, rendering them promising candidates as biomarkers. Mechanistically, circRNAs have been described as miRNA competitive inhibitors or protein decoys as transcriptional and alternative splicing modulators and, in some instances, as templates for the synthesis of functional peptides [14,15]. Dysregulated circRNA expression has been observed in a variety of disease states such as cardiovascular diseases, neurodegenerative diseases, and metabolic diseases, several of which are recognised comorbidities of ED [16].

The role of circRNAs in ED is still relatively poorly understood. Up to now, no research has systematically elucidated the mechanistic connections between particular circRNAs and the essential signalling pathways involved in ED, and no systematic attempt has been made to incorporate these regulatory networks into testable hypotheses for biomarker identification or therapeutic intervention.

While the roles of miRNAs and lncRNAs in ED have been addressed in existing reviews [17,18], circRNAs possess biological properties—resistance to exonucleolytic degradation, capacity for miRNA sponging, and tissue-specific expression—that differentiate them mechanistically from other ncRNA classes and justify a dedicated synthesis.

This review addresses two research questions: first, what direct experimental evidence currently exists for circRNA dysregulation in ED; and second, which circRNAs identified in related vascular, smooth muscle, and neuronal model systems constitute mechanistically plausible candidates for ED-specific investigation. As detailed in Section 2, only two studies have directly investigated circRNA dysregulation in ED to date; given this scarcity of direct evidence, the present work adopts a hybrid systematic narrative design rather than presenting itself as a classical systematic evidence synthesis. The systematic component (Section 2.1) establishes the boundary of direct ED-specific evidence; the narrative component (Section 2.2) is explicitly hypothesis-generating, mapping circRNAs identified in related pathway-relevant model systems onto the four principal signalling pathways involved in ED, namely, the NO/cGMP/PKG, RhoA/ROCK, JNK/c-Jun/Bak, and the TNF-α/NF-κB pathways. Within this context, we propose a mechanistic model of circRNA perturbation as a candidate component of ED aetiology, and circRNA candidates with the strongest convergent evidence are proposed as priorities for future diagnostic or therapeutic investigation [16,19].

## 2. Materials and Methods

### 2.1. Systematic Component

A structured literature search was performed in accordance with the Preferred Reporting Items for Systematic Reviews and Meta-Analyses (PRISMA) framework, (Figure 1). Three electronic databases were searched: PubMed/MEDLINE, Scopus, and the Cochrane Library. The Boolean combination ((“erectile dysfunction” OR impotence OR “erectile function” OR “penile erection” OR “sexual dysfunction”) AND (“circular RNA” OR circRNA OR “circ-RNA” OR “circular RNAs” OR circRNAs OR “circ RNAs” OR “circular non-coding RNA” OR “covalently closed RNA”)) was used. Although broader anatomical terminology could in principle identify additional studies, an additional search using terms such as “corpus cavernosum”, “cavernous smooth muscle”, “vascular erectile dysfunction”, “cavernous endothelial dysfunction”, “post-prostatectomy erectile dysfunction” and “neurogenic erectile dysfunction” confirmed that no studies using exclusively such terms met the eligibility criteria for this review. The reference lists of identified papers, as well as reviews examining ncRNAs in the context of ED were searched, with no additional records identified. No publication date restriction was applied. The most recent search was performed on 11 April 2026.

Four investigators independently screened retrieved records. Given the limited volume of directly relevant primary literature, full-text review was applied to all retrieved articles without prior title/abstract screening. Inter-rater disagreements were resolved by consensus discussion among all reviewers.

Eligibility was determined by two pre-specified criteria: (1) the study constituted original, peer-reviewed research examining the relationship between circRNAs and erectile dysfunction; (2) the manuscript was published in the English language. Both animal studies and human-based investigations were considered eligible.

Given that preliminary searching identified only two primary studies meeting eligibility criteria, a formal PROSPERO protocol registration was not submitted prior to commencement. The review was designed from the outset as a hybrid systematic narrative synthesis rather than a classical systematic review, for which PROSPERO registration is not mandated.

A completed PRISMA 2020 checklist is provided as Supplementary Figure S1 [20].

### 2.2. Narrative Component

In parallel, a narrative synthesis was conducted to identify circRNA–pathway interactions relevant to ED pathogenesis. The four pathways examined—NO/cGMP/PKG, RhoA/ROCK, JNK/c-Jun/Bak, and TNF-α/NF-κB—were selected a priori on the basis of direct experimental evidence of involvement in ED from prior non-circRNA literature and constituting of established molecular targets plausibly regulatable by circRNA-mediated miRNA sponging. Studies were identified by targeted pathway-specific searches of PubMed and Scopus combining each pathway’s principal molecular constituents (e.g., ROCK1, eNOS, NF-κB, JNK) with circRNA-related terms, supplemented by reference list screening. Studies were included if they: (i) reported experimental evidence for a direct circRNA–target interaction within one of these pathways; (ii) employed a model system with relevance to vascular endothelium, smooth muscle, or neuronal tissue; and (iii) identified a specific miRNA or downstream protein target. Studies from neoplastic models were included only where the identified axis had independent evidence of activity in non-neoplastic tissue, or where the pathway had direct ED relevance established by prior non-circRNA literature. Tier assignments were made independently by two investigators (C.D. and C.M.). Discrepancies were resolved by consensus. The tier system applies equally to circRNAs identified both during the systematic and the narrative search. Tier A is reserved for studies meeting Section 2.1 eligibility criteria. This process was iterative rather than exhaustive, consistent with the hypothesis-generating purpose of the narrative component.

The PRISMA flow diagram was applied to the systematic component of the review, to ensure methodological rigour. The extremely limited number of studies located is in itself a noteworthy finding. To better address the research question, a hybrid design (systematic and narrative component) was applied to the review, ensuring both exhaustive literature search and evidence synthesis.

## 3. Results

### 3.1. Systematic Literature Review

An overview of the circRNA–pathway interactions discussed in this review is presented in Figure 2.

The earliest study found through the systematic review is that by Huang et al. (2021) [19], which used a rat model of cavernous nerve crush injury-induced ED (CNI-ED) to analyse circRNA expression differences. Differential expression of eleven circRNAs was detected through transcriptomic analysis in the comparisons between CNI-ED and sham groups: ciRNA351, circRNA2981, circRNA2982, circRNA2983, circRNA2984, circRNA2986, circRNA3009, circRNA945, circRNA1063, ciRNA109, and ciRNA171. A competing endogenous RNA (ceRNA) network including ten of these transcripts was built, seven of which were up-regulated (ciRNA351, circRNA2981–2984, circRNA2986, circRNA3009) and three down-regulated (circRNA945, ciRNA109, ciRNA171). KEGG enrichment analysis of the ceRNA network identified downstream gene targets including Ccna2, Cxcl10, Pld1, Mapk11, and Mboat2 associated with energy metabolism, with Cxcl10 expression presumed to be regulated by the sponging of miR-27b-5p. Interestingly, three of the circRNAs identified were found to be conserved between rat and human genomes, although no clinical validation has been carried out so far. An additional constraint is that the model mainly reproduces post-radical-prostatectomy ED, thereby limiting external validity [19].

The later study, conducted by Luo et al. (2026) [21], explored circRNA aberrations in diabetes mellitus-induced erectile dysfunction (DMED) through the use of a rat model that was centred on the Hippo/Yes-associated protein 1 (YAP1) pathway. Regulation of endothelial cell proliferation and viability is a fundamental function of the Hippo signalling pathway, and its aberrant activity has been linked to vascular damage. Within the framework of DMED, an increase in glucose concentration was found to decrease the level of circPBX1, releasing miR-195-5p from sponging. The free miR-195-5p then repressed YAP1 expression, which, in turn, decreased vascular endothelial growth factor (VEGF) secretion and endothelial cell integrity. Consequently, the circPBX1/miR-195-5p/YAP1/Hippo–VEGF signalling cascade has been proposed as the molecular cascade by which glucose toxicity induces DMED, and warrants prospective clinical validation [21].

The two studies are the only primary sources explicitly exploring the circRNA–ED interaction as determined by our comprehensive review, indicating that research into this area is still in its infancy. The following sections therefore examine circRNAs with experimental evidence of involvement in four signalling cascades central to ED pathogenesis: the JNK/c-Jun/Bak, NO/cGMP/PKG, RhoA/ROCK, and TNF-α/NF-κB pathways. In the absence of tissue-specific experimental support in penile tissue, the potential for generalisation from vascular and smooth muscle cell systems to penile tissue is considered.

A three-tier evidence classification was applied to contextualise the distance of each study’s model system from human erectile physiology. Tier A denotes direct experimental evidence obtained in an ED animal model or ED-specific cell system. Tier B denotes evidence from non-penile vascular, endothelial, or smooth muscle cell models that have established pathophysiological relevance to ED. This tier includes HUVECs (human umbilical vein endothelial cells), VSMCs (human vascular smooth muscle cells), and aortic or cardiac endothelial cells, which share the fundamental contractile, oxidative, and inflammatory response programmes of cavernosal endothelium and smooth muscle. Mechanistic extrapolation from Tier B data to cavernosal physiology is considered biologically plausible but requires direct experimental confirmation. Tier C denotes evidence derived exclusively from neoplastic cell lines or neuronal tissue, where the model system shares no established pathophysiological link to penile function. Tier C findings are included where they illustrate a mechanistically conserved circRNA–miRNA–target axis that is biologically relevant to one of the four pathways examined. Extrapolation from Tier C data requires particular caution, as it represents hypothesis-generating inference rather than a mechanistic conclusion. Retaining Tier C evidence serves two purposes: it identifies specific, testable circRNA–miRNA–target candidates for future validation in cavernosal tissue, consistent with this review’s hypothesis-generating aim; and it documents the full extent of the literature searched, making explicit the boundary beyond which extrapolation to ED is unsupported.

### 3.2. The JNK/c-Jun/Bak Signalling Pathway

All circRNAs discussed in this section represent Tier C evidence, derived from neoplastic or non-penile cell models; their relevance to cavernosal apoptosis is mechanistically plausible but experimentally unconfirmed in penile tissue.

The JNK/c-Jun/Bak pathway has been specifically associated with the development of ED in two separate models [22,23]. Damage to the cavernous nerve induces activation of c-Jun N-terminal kinase (JNK) in the smooth muscle tissue of the penis, leading to the phosphorylation of c-Jun, an increase in AP-1-dependent transcription of the pro-apoptotic gene Bak, and then to apoptosis of cavernosal smooth muscle cells [23]. The role of this pathway was illustrated by Kim et al., who found that the use of SP600125, an agent that inhibits JNK, resulted in a marked recovery of intracavernosal pressure after nerve crush injury in rats, thereby implicating JNK as a promising potential therapeutic target in post-prostatectomy ED [24]. Follow-up studies where JNK inhibitors were used in conjunction with hepatocyte growth factor (HGF) therapy resulted in greater survival of corporal smooth muscle and endothelium, validating the JNK pathway as a key mediator [25]. Several circRNAs have been reported as regulators of the JNK pathway, offering mechanisms by which ncRNA dysregulation could trigger cavernosal apoptosis [26]. circUBAP2, overexpressed in lung adenocarcinoma, enhances JNK and ERK1/2 activity. Knockdown of circUBAP2 results in a reduction in JNK phosphorylation and a shift from Bcl-2/Survivin towards Bax, raising the hypothesis that circUBAP2 overexpression in cavernosal smooth muscle could similarly promote nerve injury-induced apoptosis, a possibility requiring direct confirmation in penile tissue [27]. circ-102004 facilitates JNK pathway activation along with the proliferation and migration of prostate cancer cells [28]. circMAN2B2 acts as a miR-145 sponge, derepressing both the PI3K/AKT and JNK pathways in gastric carcinoma, illustrating convergent pathway activation through a single circRNA species [29]. circLPAR3 is overexpressed in oesophageal squamous cell carcinoma, where it acts as a miR-198 sponge, a negative regulator of c-MET kinase, to promote c-MET-mediated activation of STAT3/JNK signalling [26,30]. circ-ZKSCAN1, which is overexpressed in non-small cell lung carcinoma, suppresses JNK and MAPK signalling by sponging miR-330-5p and thereby de-repressing FAM83A, a negative MAPK regulator; however, circ-ZKSCAN1 functions as an oncogene in this context—its net effect is promotion of tumour progression—and any relevance of its MAPK-dampening activity to cavernosal apoptosis protection remains entirely speculative and unconfirmed in penile tissue [31]. circ-ITCH, derived from the E3 ubiquitin ligase ITCH, which is involved in the regulation of Jun family stability, might also provide resistance against diabetic microvascular complications and the associated inflammation, thus forming a potential mechanistic connection between circRNA regulatory activity and diabetic ED [32]. The inclusion of the aforementioned circRNAs here is intended to accelerate hypothesis-driven experimental design in a pathway for which no ED-specific circRNA data currently exist, not to assert their involvement in ED.

### 3.3. The NO/cGMP/PKG Signalling Pathway

Evidence in this section is predominantly Tier B (non-penile vascular or endothelial models with established pathophysiological relevance to ED), with the exception of the Alzheimer’s disease-derived neuronal data, which constitutes Tier C.

The NO/cGMP/PKG pathway is the principal erectile signalling pathway, in which nNOS- and eNOS-derived NO activates soluble guanylyl cyclase, generating cGMP and stimulating PKG-mediated smooth muscle relaxation [7]. Disruption at any point in this cascade—through eNOS uncoupling, substrate depletion, oxidative NO scavenging, or elevated PDE5 activity—reduces cavernosal blood flow and contributes to ED [33].

Several circRNAs are known to modulate the NO/cGMP/PKG cascade in the vascular endothelium. One of these is circHMGCS1 (hsa_circ_0008621), which sequesters miR-4521, resulting in the alleviation of the inhibitory effect of miR-4521 on ARG1 expression. Given that ARG1 and eNOS both utilise L-arginine as the substrate, an increase in ARG1 activity results in a decreased substrate availability for NO synthesis, which subsequently results in reduced cGMP production and consequently diminishes smooth muscle relaxation. The circHMGCS1/miR-4521/ARG1 axis has been studied in vascular endothelial dysfunction caused by type 2 diabetes mellitus, during which circHMGCS1 overexpression elevated the expression of adhesion molecules and the production of reactive oxygen species (ROS) [34]. Considering that diabetes mellitus is among the most common aetiologies of ED, this pathway is a candidate pathway of potential therapeutic interest in DMED, pending validation in penile tissue. circRNA-RBCK1, the circRNA transcribed from the RBCK1 gene (RING finger and CCCH-type zinc finger domain-containing protein 1), acts as a molecular sponge of miR-133a in cardiac endothelial cells. miR-133a negatively regulates GTP cyclohydrolase 1 (GTPCH1). GTPCH1 catalyses the rate-limiting step in BH4 biosynthesis, and BH4 is the critical cofactor required for eNOS coupling.

In the absence of circRNA-RBCK1, GTPCH1 is suppressed, BH4 levels fall, and eNOS uncouples, with superoxide generated instead of NO, further depleting NO bioavailability and consequently reducing cGMP production [35]. Induction of AP-2α by statins led to an upregulation of circRNA-RBCK1, which in turn counteracted the inhibitory effect of miR-133a and re-established the BH4-dependent coupling of eNOS. Genetic deletion or pharmacological inhibition abolished this effect in a mouse model of heart failure [35]. Re-coupling of BH4/eNOS is critically important to ED since eNOS uncoupling is an established cause of penile vascular impairment in diabetic and elderly patients. Oscillatory shear stress, which is a haemodynamic marker of disrupted blood flow at arterial bifurcations and a well-established causative factor of endothelial dysfunction, induces the expression of circRNA-LONP2 in mouse aortic endothelial cells [36]. circRNA-LONP2 inhibits the expression of NRF2 through a miR-200a-3p/YAP1/EZH2 signalling pathway, resulting in a reduction in antioxidant defences and an increase in the production of reactive oxygen species (ROS).

The ROS generated directly scavenge NO, thereby reducing cGMP production and also oxidise BH4, leading to additional uncoupling of eNOS. This establishes a self-amplifying loop of endothelial impairment that is potentially relevant to the cardiovascular diseases often comorbid with ED [36,37]. cZNF609 functions via a different but mechanistically similar pathway: as an endogenous competitive inhibitor of miR-615-5p, it leads to the de-repression of MEF2A and the promotion of a pro-inflammatory endothelial state with increased production of reactive oxygen species and increased expression of adhesion molecules, which indirectly reduce the activity of the NO/cGMP pathway [38]. Through KEGG enrichment analysis, five circRNAs, namely novel_circ_0002311, novel_circ_0009273, novel_circ_0003012, novel_circ_0003089, and novel_circ_0001331 were found to be linked to the cGMP-PKG signal transduction pathway by ceRNA networks derived from circRNA expression profiles in the hippocampus of an Alzheimer’s disease mouse model [39].

Preliminary experimental data suggest that the novel_circ_0003012/mmu-miR-298-3p/Smoc2 axis may be involved in the pathogenesis of Alzheimer’s disease via this mechanism. While originating from neuronal rather than vascular tissue, this work suggests the existence of circRNAs with downstream targets in the PKG signalling pathway rather than the previously mentioned systemic eNOS/BH4/NO pathway, indicating that circRNA-mediated regulation of the NO/cGMP/PKG pathway is not limited to the PKG-activating step.

### 3.4. The RhoA/ROCK Signalling Pathway

The circ_Lrp6 and SHR (spontaneously hypertensive rat) aortic circRNA data represent Tier B evidence from vascular smooth muscle and hypertensive aortic tissue with established relevance to ED-associated vascular dysfunction; the hsa_circ_0001591 data are Tier C, derived from a melanoma model.

The RhoA/ROCK pathway counterbalances the NO/cGMP/PKG cascade by maintaining cavernosal smooth muscle contraction through ROCK-mediated inhibition of myosin light chain phosphatase (MLCP), sustaining MLC phosphorylation independent of intracellular calcium [7,40]. Pathological RhoA/ROCK overactivation, reversed by ROCK inhibition with fasudil in hypertensive and diabetic animal models, is a recognised driver of organic ED [40]. Oxidative stress and inflammatory mediators enhance RhoA/ROCK pathway activity resulting in interactions between this pathway and the nitric oxide (NO)/cyclic guanosine monophosphate (cGMP) pathway [41]. Circular RNA Lrp6 (circ_Lrp6) is generated from the gene encoding lipoprotein receptor-related protein 6 (LRP6) and was found by RNA sequencing in VSMCs to be the most evolutionarily conserved circRNA with multiple binding sites for microRNA-145 (miR-145) [42].

Through the sequestration of miR-145, a microRNA that directly binds to and downregulates ROCK1 mRNA, thus inhibiting proliferation and contraction of vascular smooth muscle cells, especially during hyperglycaemic states [43], circ_Lrp6 blocks the suppression of ROCK1 gene expression, promoting RhoA/ROCK pathway-induced vasoconstriction. This interaction is potentially relevant pathophysiologically to diabetic ED in which hyperactivation of the RhoA/ROCK pathway is a well-known mediator of penile smooth muscle dysfunction, although direct confirmation in cavernosal tissue is required.

In the aorta of SHRs, transcriptomic analysis uncovered three circRNA–miRNA–mRNA regulatory axes that are built around transcription factors NOTCH1, FOXO3, and STAT3 [44]. These transcription factors are recognised as regulators of RhoA/ROCK pathway activity [45,46,47].

hsa_circ_0001591 adds an additional circRNA-ROCK1 regulatory interaction. In human melanoma, this circRNA enhances cellular proliferation and invasiveness through sequestration of miR-431-5p, a microRNA with targets such as ROCK1 [48]. This circRNA has no reported expression or function in vascular, smooth muscle, or cavernosal tissue of any kind. It is presented here solely to illustrate that the miR-431-5p/ROCK1 interaction is conserved beyond the neoplastic context—not as evidence that hsa_circ_0001591 plays any role in erectile physiology. Any relevance to cavernosal smooth muscle tone is entirely speculative and would require experimental demonstration in penile tissue before it could be considered further.

Collectively, these findings suggest a candidate regulatory network in which circRNA may sustain pathological RhoA/ROCK activity in cavernosal tissue [41]. Inflammation of the vascular system is a well-established factor in the aetiology of erectile dysfunction. TNF-α, secreted by stimulated macrophages and compromised endothelium, induces vasoconstriction within the corpus cavernosum through potentiation of sympathetic- and α-adrenergic agonist-induced contractions and through the engagement of the RhoA/ROCK pathway, all of which leads to the eventual activation of the contractile proteins described in the previous paragraph [40].

### 3.5. The TNF-α/NF-κB Signalling Pathway

The circRNAs discussed in this section represent Tier B evidence, derived from vascular endothelial and smooth muscle models with established relevance to ED pathophysiology.

Concomitantly, TNF-α-induced NF-κB activation inhibits eNOS gene transcription, thus reducing NO levels and resulting in increased expression of adhesion molecules (VCAM-1, ICAM-1) and pro-inflammatory cytokines which cause vascular damage [40]. Endothelial dysfunction, defined by a reduced NO/cGMP/PKG pathway and an increased oxidative and inflammatory stress, is a pathophysiological feature of ED [37,40]. circ-Sirt1, derived from the SIRT1 gene locus, inhibits NF-κB activity in vascular smooth muscle cells (VSMCs) via a dual mechanism [49]. Within the cytoplasm, circ-Sirt1 directly sequesters NF-κB p65 through sequence-specific binding, preventing its nuclear translocation; this mechanism operates independently of the classical IKK/IκB pathway. Simultaneously, circ-Sirt1 sponges miR-132 and miR-212, de-repressing SIRT1 expression. Elevated SIRT1 subsequently deacetylates and inactivates nuclear p65, providing a secondary mode of transcriptional repression.

Downregulation of circ-Sirt1, observed in conditions of oxidative stress and atherosclerosis, removes this brake on TNF-α-stimulated NF-κB activation and may, by analogy, impair vascular homeostasis in erectile tissue, pending direct experimental confirmation [49]. circ-RELL1 is a proinflammatory circRNA in the endothelium. In oxidised low-density lipoprotein (oxLDL)-treated HUVECs, circ-RELL1 is upregulated and acts as a miR-6873-3p sponge. This sequestration de-represses MyD88 (myeloid differentiation primary response protein 88), a critical adaptor in Toll-like receptor and interleukin-1 receptor signalling cascades, leading to canonical NF-κB pathway activation and upregulation of adhesion molecules ICAM-1 and VCAM-1 [50].

This mechanism is of potential relevance to atherogenic ED, in which the accumulation of oxidised LDL in penile vascular beds causes endothelial damage.

A summary of the circRNAs discussed above is presented in Table 1.

## 4. Discussion

The data presented favour a model in which circRNA misexpression plays a role in ED through a number of mechanistically related pathways. Systemic inflammatory stress indicated by increased levels of C-reactive protein (CRP), neutrophil–lymphocyte ratio (NLR), and platelet–lymphocyte ratio (PLR) has been prospectively associated with ED [51,52,53], and inflammatory cytokine signatures in men with ED differ from those of controls [54]. Current evidence indicates that inflammation contributes to ED primarily through endothelial injury, reduction in NO bioavailability, and reduced corpus cavernosal blood flow [55,56]. Consequently, the circRNAs that modulate the NF-κB/TNF-α axis (notably circ-Sirt1 as a suppressor and circ-RELL1 as an amplifier) are well-positioned to determine the balance of this inflammatory–vascular interaction. While the role of circRNAs in inflammation has been comprehensively reviewed [57,58,59], how specific circRNA–pathway interactions shape ED pathophysiology has not previously been addressed, and constitutes the central focus of this review. Numerous studies have been conducted in the pursuit of stable molecular indicators of ED [60,61,62], with non-coding RNAs emerging as especially promising candidates [63]. Although endothelial damage is the well-recognised fundamental pathogenic process through which cardiovascular risk factors contribute to ED, there is a notable lack of systematic investigation of circRNAs as endothelial-specific markers in ED [64]. The circRNAs identified in this review, circHMGCS1, circRNA-RBCK1, circ-Sirt1, circ_Lrp6, and circPBX1, among others, share structural properties generally associated with potential biomarkers: a covalently closed structure conferring enhanced stability [13], expression in a tissue- and disease-specific manner, and the theoretical potential for detectability in circulating biological fluids such as serum and plasma [16]. However, none of these five circRNAs has been measured in human blood, plasma, or penile tissue in any study to date, and direct evidence for their role in ED remains limited to two animal studies. Accordingly, they should be regarded strictly as candidates for future biomarker and therapeutic target validation, not as established or even provisionally validated biomarkers or therapeutic targets. It should also be noted that miRNA sponging efficiency depends on whether the circRNA is expressed at sufficient concentrations to sequester its target miRNA—a stoichiometric constraint that has not been addressed by any of the studies discussed in this review [13].

### 4.1. Limitations

Several important limitations apply to the models proposed above. This review does not provide an exhaustive analysis of all pathways involved in ED; other axes, such as the renin–angiotensin system [65] and the Sonic Hedgehog (Shh) pathway [66,67], are also involved in the pathophysiology of ED and may, as yet, contain circRNA regulatory interactions that have not been characterised. For the pathways examined, direct evidence of circRNA involvement in ED has been demonstrated in only two studies, both relying on rat models [19,21]. The remaining circRNAs discussed were characterised in non-penile vascular, smooth muscle, or neoplastic cell models. Therefore, any extrapolation to cavernosal tissue should be treated as a hypothesis until direct in situ data are available.

It must be emphasised that the majority of the circRNA candidates discussed in this review are supported exclusively by Tier B or Tier C evidence—that is, evidence from non-penile vascular, smooth muscle, or neoplastic cell models. The step from “mechanistically plausible” to “specifically relevant to ED” has not been experimentally demonstrated for any of these candidate circRNAs, and the review does not claim otherwise. Each such candidate should be read as a hypothesis to be tested, not as an established mechanism.

A structured critical appraisal of the two primary studies reveals important constraints. Huang et al. [19] employed a bilateral cavernous nerve crush rat model with a sample size of twelve animals (six per group), which is small by clinical standards, though appropriate for a pilot transcriptomic study in this experimental system. This model primarily reproduces neurogenic post-radical-prostatectomy ED and may not generalise to vasculogenic, hormonal, or psychogenic ED subtypes. Differential circRNA expression was reported across the full transcriptome without functional validation of any individual transcript; the ceRNA network was constructed computationally and has not been experimentally confirmed. Three of the network circRNAs are conserved between the rat and human genomes, but none has been detected or quantified in human penile tissue, and no circulating biomarker measurements were reported. In the case of Luo et al. [21], the sample size is not reported in the available record. The circPBX1/miR-195-5p/YAP1 axis was supported by in vitro rescue experiments in penile cavernous endothelial cells and by adenovirus-mediated in vivo knockdown experiments in DMED rats, providing functional evidence of a causal role; this makes Luo et al. the methodologically stronger of the two primary studies. However, clinical validation in human DMED cohorts—including measurement of circPBX1 in serum, plasma, or penile tissue from patients—has not been performed, and no formal risk-of-bias assessment of either study was conducted as part of this review.

Several methodological limitations of this review require explicit statement. The direct evidence base is sparse: two primary studies, both in rat models, constitute the entirety of Tier A evidence; neither measures circulating biomarker levels nor includes human tissue, meaning no circRNA identified here qualifies as a validated biomarker or therapeutic target under any standard definition. The majority of candidates rely on Tier B or Tier C extrapolation. Tier B inference from HUVECs or VSMCs rests on shared pathway biology, but the corpus cavernosum is haemodynamically distinct and its circRNA expression profile may differ substantially. Tier C extrapolation from neoplastic models is more tenuous still, since malignant transformation fundamentally reprogrammes ceRNA networks. Finally, no formal risk-of-bias assessment was performed; the appropriate instrument for animal studies of this kind is the SYRCLE tool, whose application was beyond the scope of this hybrid synthesis [68].

Crucially, the molecular mechanisms of ED are not independent: RhoA/ROCK overactivity can be caused by the TNF-α/NF-κB pathway, eNOS inhibition by ROCK reduces NO bioavailability, and JNK activation promotes the overproduction of ROS that uncouple eNOS. The therapeutic relevance of this convergence is supported indirectly: pharmacological inhibition of the downstream Rho-kinase/LIM-kinase/cofilin axis reduces corporal fibrosis and preserves erectile function in aged animal models [69]. This establishes biological plausibility for circRNA-based modulation of this pathway as a candidate future therapeutic strategy; however, this evidence concerns small-molecule kinase inhibition rather than circRNA modulation specifically, and no circRNA-based intervention in this pathway has yet been tested in any ED model. Multiple circRNAs are involved in multiple pathways simultaneously; for example, circRNA-LONP2 suppresses NRF2 expression through the YAP1/EZH2 axis, enabling NF-κB-induced inflammation in addition to its direct effects on NO bioavailability [36]. These cross-pathway interactions suggest that circRNA-mediated regulatory effects may be substantially greater than analysis of any single pathway in isolation would predict. Ultimately, the clinical manifestation of ED converges on a single functional question: whether cavernosal smooth muscle can attain the relaxation required for sufficient corporal engorgement.

The most important gap this review identifies is the complete absence of circRNA profiling data from human cavernosal tissue in patients with ED. Without such research, the potential of circRNAs as possible biomarkers or therapeutic targets cannot be ascertained. Therefore, further research is needed to confirm the claims made in this review.

### 4.2. Future Directions

The findings of this review point to a clear hierarchy of experimental priorities. First, human cavernosal transcriptomic profiling combining RNA-seq with dedicated circRNA detection pipelines is required to determine which candidate circRNAs are expressed in penile tissue and whether their expression is altered in ED patients. Second, cell-type-specific validation using primary penile endothelial and smooth muscle cells is necessary before any mechanistic claim can be considered ED-relevant. Third, circulating biomarker feasibility studies measuring serum or plasma levels of candidate circRNAs in well-phenotyped ED cohorts would address the clinical translation gap directly. Fourth, functional perturbation experiments in ED-relevant in vitro and in vivo systems are needed to move from mechanistic plausibility to causal evidence. Finally, the stoichiometric requirements for effective miRNA sponging must be addressed quantitatively, as sufficient endogenous circRNA concentration to sequester target miRNAs is a prerequisite for both biomarker utility and therapeutic relevance.

Therapeutically, circRNA resistance to exonucleolytic degradation confers stability [13] but complicates conventional antisense approaches; siRNA directed against the back-splice junction is a more appropriate modality [13,15]. Overexpression via adenoviral vectors has already been demonstrated in the primary ED literature—Luo et al. used adenovirus-mediated knockdown of circPBX1 in DMED rats with measurable erectile improvement [21], and Li et al. applied the same approach to modulate circRNA-RBCK1 in a vascular model [35], establishing proof of concept for viral delivery of circRNA interventions. Intracavernosal injection is an established clinical route [5,6] whose feasibility for molecular interventions has been demonstrated [24,25], making locally delivered therapies anatomically conceivable pending systematic preclinical evaluation.

Clinically, cross-sectional studies measuring circRNA levels in serum or plasma from men with and without ED—following the design of existing inflammatory biomarker studies in this population [51,52,53,54]—would establish whether candidate levels differ between groups. Prospective longitudinal designs following men with vasculogenic risk factors over time would determine whether circRNA changes precede erectile decline [60,61,62]. Treatment-response studies measuring circRNA dynamics before and after PDE5 inhibitor therapy or lifestyle intervention [5,6] would establish whether these profiles are modifiable and track clinical improvement. None of these designs currently exists in the literature; implementing them should be a priority in this nascent field.

## 5. Conclusions

Erectile dysfunction is a multifactorial molecular disease involving multiple interacting pathways. This review has mapped circRNA regulatory interactions across four signalling pathways central to ED pathogenesis: NO/cGMP/PKG, RhoA/ROCK, JNK/c-Jun/Bak, and TNF-α/NF-κB pathways. To date, only two studies have explicitly examined the connection between circRNA and ED: Huang et al. [19] in CNI-ED and Luo et al. [21] in DMED. Nevertheless, the circRNAs implicated in ED-relevant signalling pathways are diverse in both tissue origin and cell-type specificity. Considering their structural integrity and the cell-type-specific gene expression, these molecules represent candidates warranting future biomarker and therapeutic target validation; none can currently be considered established, given that direct evidence remains limited to two animal studies with no human tissue or circulating validation.

## Figures and Tables

**Figure 1:**
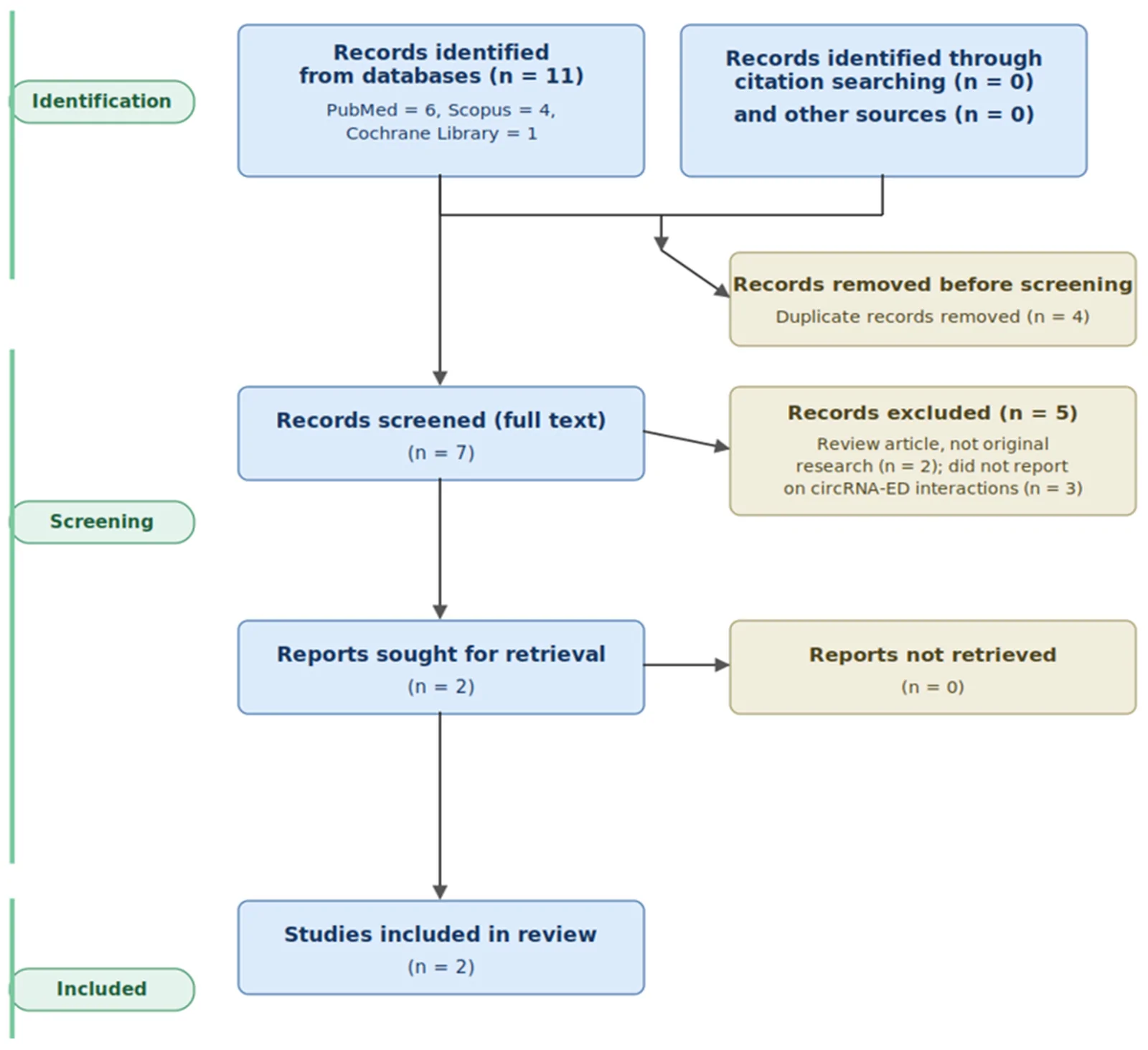
PRISMA 2020 flow diagram of the literature search procedure.

**Figure 2:**
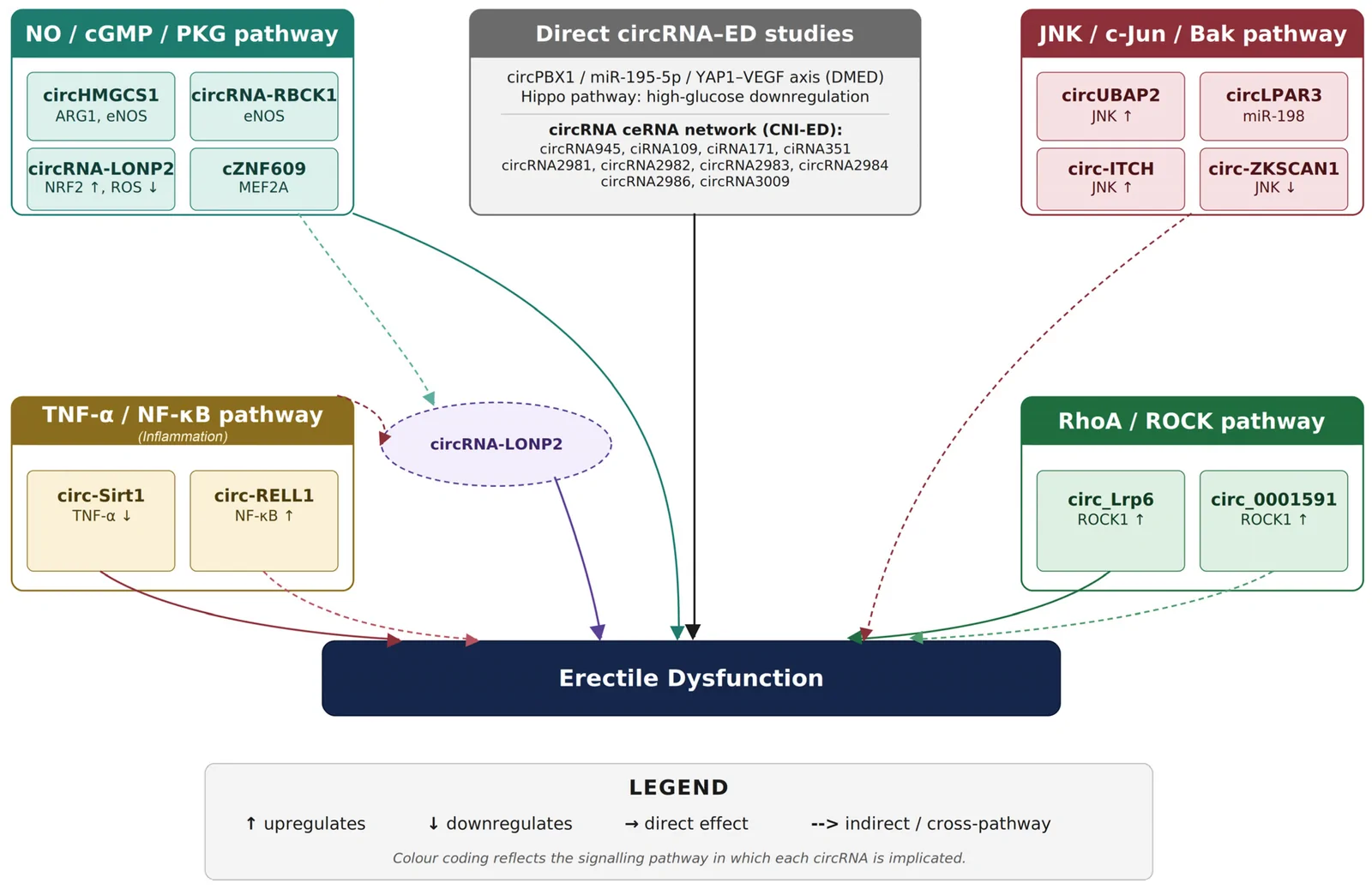
Candidate circRNA interactions in signalling pathways implicated in erectile dysfunction. Four principal ED-associated signalling cascades are shown (NO/cGMP/PKG, JNK/c-Jun/Bak, RhoA/ROCK, TNF-α/NF-κB) alongside the circRNA species documented to modulate each. The central panel depicts circRNAs with direct experimental evidence in ED models. Arrows indicate direction of effect (↑ upregulation; ↓ downregulation). The dashed line connecting NO/cGMP/PKG and TNF-α/NF-κB axes via circRNA-LONP2 illustrates cross-pathway regulatory interactions. For visual clarity, Figure 2 presents a representative selection of the most discussed circRNA candidates in each pathway. The complete set of circRNA candidates discussed in this review, including all Tier B and Tier C entries, is provided in Supplementary Table S1. ARG1, arginase 1; BH4, tetrahydrobiopterin.

**Table 1:** Summary of circRNA candidates discussed in this review, organised by evidence tier. Tier A: direct experimental evidence in an ED animal model or ED-specific cell system. Tier B: evidence from non-penile vascular, endothelial, or smooth muscle cell models with established pathophysiological relevance to ED. Tier C: evidence derived exclusively from neoplastic cell lines or neuronal tissue, where extrapolation to cavernosal physiology requires particular caution and validation. For full mechanistic detail, model systems, and miRNA targets, see Supplementary Table S1.

Evidence Tier	Pathway	circRNA	Key Mechanism
**Tier A**	Hippo/YAP1–VEGF	*circPBX1*	Hyperglycaemia ↓ circPBX1 → ↑ free miR-195-5p → ↓ YAP1/VEGF → impaired cavernosal angiogenesis
**Tier A**	ceRNA network	*ciRNA351/circRNA2981–2984, 2986/circRNA3009*	Differentially expressed in CNI-ED rat cavernosum; ceRNA network linked to energy metabolism; three transcripts conserved with human genome
**Tier B**	NO/cGMP/PKG	*circHMGCS1*	↑ ARG1 → L-arginine competition with eNOS → ↓ NO/cGMP (T2DM human vascular endothelial cells)
**Tier B**	NO/cGMP/PKG	*circRNA-RBCK1*	Sponges miR-133a → ↑ GTPCH1/BH4 → eNOS recoupling → restored NO (mouse cardiac endothelial cells)
**Tier B**	NO/cGMP/PKG + TNF-α/NF-κB	*circRNA-LONP2*	Induced by oscillatory shear stress → ↑ ROS, NF-κB; cross-pathway amplifier (mouse aortic endothelial cells)
**Tier B**	NO/cGMP/PKG	*cZNF609*	Sponges miR-615-5p → ↑ MEF2A → ↓ NO pathway activity (HUVECs)
**Tier B**	RhoA/ROCK	*circ_Lrp6*	Sponges miR-145 → ↑ ROCK1 → ↑ VSMC contraction; most relevant in hyperglycaemic states (human VSMCs)
**Tier B**	RhoA/ROCK	*rno_circRNA_0005818/rno_circRNA_0009197*	SHR aortic tissue; axes built around NOTCH1, STAT3, and FOXO3; recognised RhoA/ROCK regulators
**Tier B**	TNF-α/NF-κB	*circ-Sirt1*	Dual NF-κB suppressor: cytoplasmic p65 sequestration + miR-132/212 sponging → ↑ SIRT1 (VSMCs)
**Tier B**	TNF-α/NF-κB	*circ-RELL1*	↑ by oxLDL → sponges miR-6873-3p → ↑ MyD88/NF-κB → ↑ ICAM-1/VCAM-1 (HUVECs)
**Tier C**	cGMP/PKG	*novel_circ_0003012 (and 4 related)*	ceRNA network linked to cGMP-PKG pathway via KEGG enrichment; Alzheimer’s disease mouse hippocampus
**Tier C**	JNK/c-Jun/Bak	*circUBAP2, circ-102004, circMAN2B2, circLPAR3, circ-ZKSCAN1, circ-ITCH*	Various neoplastic models; mechanistically plausible but entirely unconfirmed in penile tissue
**Tier C**	RhoA/ROCK	*hsa_circ_0001591*	Sponges miR-431-5p → ↑ ROCK1 (melanoma); included solely to illustrate conservation of miR-431-5p/ROCK1 interaction; no cavernosal or vascular data

## Data Availability

No new data were created or analyzed in this study. Data sharing is not applicable to this article.

## Supplementary Materials

The following supporting information can be downloaded at: https://www.mdpi.com/article/10.3390/cimb48100994/s1.
