# Supplementary material for: CircRNAs and Erectile Dysfunction: A Hybrid Review of Possible Interactions
Source: Curr Issues Mol Biol. 2026 Sep 26;48(10):994. doi: 10.3390/cimb48100994 (PMC13703438; doi:10.3390/cimb48100994)
Supplement: Supplementary file 1 [file cimb-48-00994-s001.zip › Supplementary Table S1.pdf]

Supplementary Table S1.

Summary of circRNA candidates discussed in this review, classified by associated signalling pathway, experimental model system, miRNA target(s), proposed mechanistic role in ED pathogenesis, and evidence tier. Tier A: direct experimental evidence in an ED animal model or ED-specific cell system. Tier B: evidence from non-penile vascular, endothelial, or smooth muscle cell models with established pathophysiological relevance to ED (e.g. HUVECs, VSMCs, aortic endothelial cells, cardiac endothelial cells). Tier C: evidence derived exclusively from neoplastic cell lines or neuronal tissue, where extrapolation to cavernosal physiology requires particular caution and validation. DMED, diabetes mellitus-associated erectile dysfunction; CNI-ED, cavernous nerve injury-induced erectile dysfunction; HUVECs, human umbilical vein endothelial cells; VSMCs, vascular smooth muscle cells; oxLDL, oxidised low-density lipoprotein; SMC, smooth muscle cell; T2DM, type 2 diabetes mellitus; BH4, tetrahydrobiopterin.

circRNA	Pathway	Model system	miRNA target(s)	Proposed mechanism in ED context	Evidence tier
			Direct ED evidence		
circPBX1 [20]	Hippo/YAP1–VEGF	Rat DMED model; penile cavernous endothelial cells	miR-195-5p	Hyperglycaemia ↓ circPBX1 → free miR-195-5p represses YAP1 → ↓ VEGF → impaired endothelial integrity and angiogenesis	**Tier A**
ciRNA351 / circRNA2981–2984, circRNA2986 / circRNA3009 [19]	ceRNA network (CNI-ED)	Rat cavernous nerve crush model	miR-27b-5p (Cxcl10)	Differentially expressed in CNI-ED; ceRNA network linked to energy metabolism genes (Ccna2, Cxcl10, Pld1, Mapk11); three transcripts conserved with human genome	**Tier A**
			NO/cGMP/PKG pathway		
circHMGCS1 (hsa_circ_0008621) [33]	NO/cGMP/PKG	Human vascular endothelial cells (T2DM model)	miR-4521	Sequesters miR-4521 → ↑ ARG1 → L-arginine competition with eNOS → ↓ NO → ↓ cGMP; of potential relevance to DMED	**Tier B**
circRNA-RBCK1 [34]	NO/cGMP/PKG	Mouse cardiac endothelial cells; heart failure model	miR-133a	Sponges miR-133a → ↑ GTPCH1 → ↑ BH4 → eNOS recoupling → restored NO; relevant to penile vascular impairment in diabetes and ageing	**Tier B**
circRNA-LONP2 [35]	NO/cGMP/PKG + TNF-α/NF-κB	Mouse aortic endothelial cells (oscillatory shear stress model)	miR-200a-3p	Induced by oscillatory shear stress → inhibits NRF2 via YAP1/EZH2 → ↑ ROS → NO scavenging + NF-κB activation; cross-pathway amplifier of endothelial dysfunction	**Tier B**
cZNF609 [37]	NO/cGMP/PKG (indirect)	HUVECs	miR-615-5p	Sponges miR-615-5p → ↑ MEF2A → pro-inflammatory endothelial state → ↓ NO pathway activity	**Tier B**
novel_circ_0003012 (and 4 related) [38]	cGMP/PKG (downstream)	Alzheimer's disease mouse hippocampus (neuronal)	mmu-miR-298-3p (Smoc2)	ceRNA network linked to cGMP-PKG pathway via KEGG enrichment; suggests circRNA regulation extending to the PKG-activating step, not only eNOS/BH4	**Tier C**
			JNK/c-Jun/Bak pathway		
circUBAP2 [26]	JNK/c-Jun/Bak	Lung adenocarcinoma cells	Not fully characterised	Overexpression → ↑ JNK + ERK1/2 phosphorylation → pro-apoptotic shift (Bcl-2/Survivin → Bax); extrapolated to cavernosal SMC apoptosis after nerve injury	**Tier C**
circ-102004 [27]	JNK/c-Jun/Bak	Prostate cancer cells	Not characterised	Facilitates JNK pathway activation; anatomical proximity to prostatectomy site noted, unverified functionally.	**Tier C**
circMAN2B2 [28]	JNK + PI3K/AKT	Gastric carcinoma	miR-145	Sponges miR-145 → derepresses JNK and AKT in two independent cancer models; illustrates convergent multi-pathway activation through a single circRNA species	**Tier C**
circLPAR3 [29]	JNK/STAT3	Oesophageal squamous cell carcinoma	miR-198	Sponges miR-198 → ↑ c-MET → STAT3/JNK activation; relevant if JNK-mediated cavernosal apoptosis extends beyond nerve injury models	**Tier C**

circRNA	Pathway	Model system	miRNA target(s)	Proposed mechanism in ED context	Evidence tier
circ-ZKSCAN1 [30]	JNK/MAPK (suppressor)	Non-small cell lung carcinoma	miR-330-5p	Oncogene; sponges miR-330-5p, de-repressing FAM83A and suppressing JNK/MAPK, promoting tumour progression; MAPK-dampening effect is mechanistically real but serves cancer promotion; speculative and unconfirmed relevance to cavernosal apoptosis	**Tier C**
circ-ITCH [31]	JNK (Jun stability)	Tumour and non-tumour models	Multiple	Regulates Jun family stability; proposed protection against diabetic microvascular inflammation; potential mechanistic connection to DMED	**Tier C**
			RhoA/ROCK pathway		
circ_Lrp6 [41]	RhoA/ROCK	Human vascular smooth muscle cells (VSMCs)	miR-145	Sequesters miR-145 → ↑ ROCK1 → ↑ VSMC contraction; most relevant in hyperglycaemic states; established pathophysiological relevance to diabetic vascular dysfunction, with plausible extension to DMED	**Tier B**
rno_circRNA_0005818 [43]	RhoA/ROCK	Spontaneously hypertensive rat (SHR) aortic vascular tissue	miR-615 (→ NOTCH1); miR-10b-5p (→ STAT3)	Sponges miR-615 and miR-10b-5p, de-repressing NOTCH1 and STAT3 (recognised regulators of RhoA/ROCK pathway); candidate contributor to hypertension-associated vascular smooth muscle dysfunction relevant to organic ED	**Tier B**
rno_circRNA_0009197 [43]	RhoA/ROCK	Spontaneously hypertensive rat (SHR) aortic vascular tissue	miR-509-5p (→ FOXO3)	Loss of rno_circRNA_0009197 reduces sponging of miR-509-5p, suppressing FOXO3, a negative regulator of RhoA/ROCK activity; consistent with RhoA/ROCK pathway overactivation in hypertension-associated ED	**Tier B**
hsa_circ_0001591 [47]	RhoA/ROCK	Human melanoma cells	miR-431-5p	Sponges miR-431-5p → ↑ ROCK1 in melanoma; miR-431-5p/ROCK1 axis conserved(no data on vascular/smooth muscle/cavernosal tissue)	**Tier C**
			TNF-α/NF-κB pathway		
circ-Sirt1 [48]	TNF-α/NF-κB	VSMCs; atherosclerosis models	miR-132, miR-212	Dual mechanism: cytoplasmic sequestration of NF-κB p65 + sponges miR-132/212 → ↑ SIRT1 → p65 deacetylation; ↓ expression in oxidative stress → ↑ NF-κB in penile vasculature	**Tier B**
circ-RELL1 [49]	TNF-α/NF-κB	HUVECs (oxLDL model)	miR-6873-3p	↑ by oxLDL → sponges miR-6873-3p → ↑ MyD88 → NF-κB activation → ↑ ICAM-1/VCAM-1; of potential relevance to atherogenic ED (validation in cavernosal tissue needed)	**Tier B**
